# The Incidence of IgG4-Related Disease in Slovenia—Single-Centre Experience

**DOI:** 10.3390/biomedicines13092281

**Published:** 2025-09-17

**Authors:** Alojzija Hočevar, Aleš Grošelj, Gregor Hawlina, Matic Koželj, Andrej Škoberne, Jože Pižem, Vesna Jurčić

**Affiliations:** 1 Department of Rheumatology, University Medical Centre Ljubljana, Vodnikova Cesta 62, 1000 Ljubljana, Slovenia; 2 Faculty of Medicine, University of Ljubljana, 1000 Ljubljana, Slovenia; ales.groselj@hotmail.com (A.G.); gregor.hawlina@gmail.com (G.H.); andrej.skoberne@kclj.si (A.Š.); 3 Department of Otorhinolaryngology and Cervicofacial Surgery, University Medical Centre Ljubljana, 1000 Ljubljana, Slovenia; 4 Eye Hospital, University Medical Centre Ljubljana, 1000 Ljubljana, Slovenia; 5 Department of Gastroenterology, University Medical Centre Ljubljana, 1000 Ljubljana, Slovenia; kozelj.matic@gmail.com; 6 Department of Nephrology, University Medical Centre Ljubljana, 1000 Ljubljana, Slovenia; 7 Institute of Pathology, Faculty of Medicine, University of Ljubljana, 1000 Ljubljana, Slovenia; joze.pizem@mf.uni-lj.si (J.P.); vesnajurcic@hotmail.com (V.J.)

**Keywords:** IgG4-related disease, epidemiology, demographic, incidence rate

## Abstract

**Background:** Data on the incidence of IgG4-related disease (IgG4-RD) are scarce. Our aim was to determine the incidence of IgG4-RD in a well-defined region. Methods: This retrospective study covered the Ljubljana region over the period from January 2012 to December 2024. A review of cases diagnosed with IgG4-RD was performed at several departments of the University Medical Centre Ljubljana—an integrated secondary/tertiary university teaching hospital (rheumatology, nephrology, angiology, gastroenterology, abdominal surgery, ENT surgery, ophthalmology). While IgG4-RD cases at the Department of Rheumatology were collected prospectively, potential cases at other departments were retrieved by searching electronic medical database for the keyword “IgG4”. In addition, the Institute of Pathology, Faculty of Medicine, University of Ljubljana, provided a list of patients with histological features consistent with IgG4-RD. Year-specific incidence rates and an average incidence rate over the 13-year period were determined. Clinical features of patients were analysed. **Results:** During the observation period, 58 cases of IgG4-RD were diagnosed. Of these, 35 patients were residents of the Ljubljana region, which had an average adult population of 541,600. The estimated average annual incidence rate of IgG4-RD was 5.0 per million (95% confidence interval: 3.5; 6.9), with year-specific incidence rates fluctuating between 1.8 and 9.3 per million adults. The cases were stratified into four phenotypic categories: pancreato-hepato-biliary (17%), retroperitoneal fibrosis-aortitis (43%), head and neck-limited (14%), and Mikulicz syndrome with systemic involvement (26%). **Conclusions:** The average annual incidence rate of IgG4-RD was 5 per million adults, with the retroperitoneal fibrosis-aortitis phenotype predominating in our cohort.

## 1. Introduction

IgG4-related disease (IgG4-RD) is a systemic autoimmune disease characterised by distinct tumefactive fibroinflammatory lesions that can affect various organs or body regions, leading to a heterogeneous clinical presentation. It is commonly accompanied by elevated serum IgG4 levels. Diagnosis is based on the synthesis of clinical, imaging, histopathological and laboratory findings. Typical histopathological features include a dense lymphoplasmacytic infiltrate enriched with IgG4-positive plasma cells, storiform fibrosis, and obliterative phlebitis [1,2,3].

More than 20 years since its recognition, epidemiological data on IgG4-RD remain very limited. The common perception that IgG4-RD is a rare condition may be misleading and could reflect underrecognition, the diversity of clinical presentations and the disease’s relatively slow progression. Two Japanese studies that assessed disease epidemiology using questionnaires sent to hospitals reported an annual incidence rate of 2.8 to 10.8 cases per million [4,5], and a prevalence of 62 per million subjects [5]. The disease onset peaked in the sixth to seventh decade of life, with a male-to-female ratio of 1.3 [5]. More recently, a study from the United States analysing an administrative claims database for the period 2015–2019 reported IgG4-RD incidence rates ranging from 7.8 to 13.9 per million person-years, a prevalence of 53 per million persons, and a male-to-female ratio of 0.74 [6].

There are no data on the incidence of IgG4-RD in Slovenia or other European countries. Therefore, the aim of our study was to retrospectively estimate, for the first time, the incidence rate of the disease in a well-defined statistical region of the country and to analyse the clinical features of our patients.

## 2. Methods

### 2.1. Setting

This retrospective observational study was conducted at the University Medical Centre (UMC) Ljubljana, Slovenia, in collaboration with the Institute of Pathology, Faculty of Medicine, University of Ljubljana, Slovenia. The study covered a 13-year period from January 2012 to December 2024.

UMC Ljubljana is the only referral hospital in the Ljubljana region and one of two tertiary medical and teaching hospitals in the country. Given the rarity and complexity of IgG4-RD, these patients are generally managed at tertiary-level institutions. Although the diagnosis of IgG4-RD can be suspected by various subspecialists, rheumatologists are commonly involved in both the diagnostic process and the long-term management of the disease. In the present study, several departments of UMC Ljubljana participated, including rheumatology, nephrology, angiology, gastroenterology, abdominal surgery, ENT surgery and ophthalmology.

The Institute of Pathology processes and analyses most biopsies performed in the Ljubljana region and provides consultation services and second opinions when needed for pathology departments in other hospitals across the country, including Slovenia’s second tertiary centre, UMC Maribor.

### 2.2. Patient Ascertainment

Patients with IgG4-RD were collected prospectively at the Department of Rheumatology. However, at other participating departments, potential cases were identified by searching the electronic medical database for the keyword “IgG4”. The medical records of all retrieved potential IgG4-RD cases were examined by a single assessor (AH).

To avoid underreporting due to potential cases diagnosed at other hospitals outside the Ljubljana region, a list of patients with a biopsy showing a histological pattern compatible with IgG4-RD was obtained from the Institute of Pathology for the same observation period. All suspected biopsies and reports were re-analysed by a single experienced pathologist (VJ).

To determine the incidence rate of IgG4-RD, we included all adult patients (aged ≥18 years) who were diagnosed with IgG4-RD for the first time during the study period and who were residents of the Ljubljana region at the time of diagnosis.

The Ljubljana region had an average adult population of 541,600 over the 13-year period, representing more than a quarter of Slovenia’s entire population [7]. The population of the Ljubljana region by study year is shown in Table 1. More than 95% of residents are Caucasians of Slavic origin (Source: Statistical Office of the Republic of Slovenia, Department of Demographic and Social Statistics [7]).

### 2.3. Characteristics of IgG4-RD at Diagnosis

Patient demographic data (age, gender), smoking habits, comorbidities and baseline IgG4-RD features—including symptom duration, organ involvement, laboratory results (serum IgG4 level, serum gammaglobulin level, complement level, blood eosinophil count), histopathological findings in cases where a biopsy was performed and initial treatment used—were extracted from medical records.

Next, patients were stratified based on the predominant pattern of IgG4-RD organ involvement into one of the four clinical phenotypes: pancreato-hepato-biliary disease, retroperitoneal fibrosis and/or aortitis, head and neck-limited disease, and Mikulicz syndrome with systemic involvement [8].

Finally, fulfilment of the 2019 ACR/EULAR IgG4-RD classification criteria [9] was assessed.

### 2.4. Statistical Analysis

The annual incidence rate for each year was calculated using the number of new IgG4-RD cases observed annually as the numerator and the adult population of the Ljubljana region for the same observation year as the denominator. A 95% confidence interval (CI) was calculated. In addition, an average annual incidence rate for the 13-year period was determined.

Descriptive statistics were used to characterise the IgG4-RD cohort. Results were expressed as medians and interquartile ranges (IQRs) for non-normally distributed variables and as means with standard deviations (SD) for normally distributed metric variables. Categorical variables were presented as absolute numbers and percentages.

## 3. Results

### 3.1. Incidence Rate of IgG4-RD

Over the 13-year study period, 58 cases of IgG4-RD were diagnosed. At the time of diagnosis, 35 out of 58 patients were residents of the Ljubljana region. The estimated average annual incidence rate of IgG4-RD during this period was 5.0 cases per million adults (95% confidence interval: (CI) 3.5; 6.9), with year-specific incidence rates fluctuating from 1.8 (95% CI: 0.05; 10.0) to 9.3 (95% CI: 2.9; 21.2) cases per million adults. Table 1 shows the number of diagnosed IgG4-RD cases and the estimated incidence rates of IgG4-RD by year. As the Slovenian population is demographically homogenous, we assume that these incidence figures are representative of the entire country.

### 3.2. Characteristics of IgG4-RD at Diagnosis

The median age at diagnosis was 64 years (53; 68). Eight out of 35 patients (22.9%) were younger than 50 years, 20 patients (57.1%) were aged 50 to 69 years, and 7 patients (20.0%) were aged 70 years or older. Twenty-six out of 35 patients (74.3%) were male (male-to-female ratio: 2.9), and 18 of 32 (56.3%) patients were ever smokers. Seven patients had a history of diabetes mellitus, seven had asthma, and six had been treated for hypothyroidism (diagnosed with Hashimoto’s thyroiditis). The median (IQR) duration of symptoms attributed to IgG4-RD before diagnosis was 6 months (3; 14), ranging from 1 to 120 months. Table 2 presents the characteristics of our IgG4-RD cohort.

The average number of organs or regions affected at diagnosis was two (ranging from one to five). Regarding IgG4-RD phenotypes, 6 (17.1%) patients had pancreato-hepato-biliary disease, 15 (42.8%) had retroperitoneal fibrosis and/or aortitis, 5 (14.3%) head and neck-limited disease, and 9 (25.7%) had Mikulicz syndrome with systemic involvement. Using the recently proposed classification into predominantly proliferative or predominantly fibrotic IgG4-RD phenotypes, 22 (62.9%) patients were classified as proliferative and 13 (37.1%) as fibrotic phenotype.

**Figure 1:**
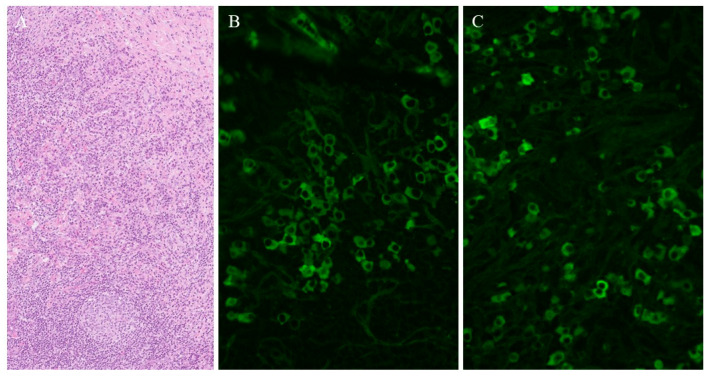
Orbital pseudotumor—IgG4-RD. Panel (**A**): Dense inflammatory infiltrate composed of plasma cells, lymphocytes and eosinophils. There is a reactive lymphatic follicle (bottom) and sclerotic stroma (upper right corner) (haematoxylin and eosin, 200× original magnification). Panel (**B**): IgG-positive plasma cells (direct immunofluorescence, 400× original magnification). Panel (**C**): IgG4-positive plasma cells. The majority (85%) of IgG-positive cells are positive for IgG4 (direct immunofluorescence, 400× original magnification).

A total of 31 out of 35 patients underwent organ biopsy, and histopathological features were consistent with IgG4-RD in 28 cases (90.3%)—reported as definite in 23 patients and probable in 5 patients. Figure 1 shows an example of histological and immunostaining findings in a patient with IgG4-RD. Serum IgG4 levels were measured in 28 patients and were elevated in 23 (82.1%) cases. Other laboratory results are presented in Table 2.

The 2019 ACR/EULAR IgG4-RD classification criteria were fulfilled in 26 patients (74.3%). Among the nine patients who did not meet the classification criteria, four had not undergone organ biopsy and two had non-informative biopsy results. One patient, who scored 19 points, did not have a determined serum IgG4 level, while the remaining two patients had organ involvement that was not scored or included in the classification criteria.

A total of 24 patients (68.6%) received systemic therapy: all 24 were treated with a systemic glucocorticoid, and 13 patients additionally received rituximab. Surgery was the only treatment in 8 patients, while 3 patients were observed only.

## 4. Discussion

Our study expands the understanding of epidemiological characteristics of IgG4-RD. It shows that in the European Slavic population, IgG4-RD is relatively rare, with an estimated annual incidence of 5 cases per million, and that disease is more prevalent in males and in the age group of 50 to 70 years. The lower incidence rate compared to that reported recently by an American research group [6] might be due to differences in ethnic composition of the investigated population, as residents of our study region are almost exclusively Caucasians of Slavic origin. In comparison, two-thirds of patients were Caucasians in the American study.

IgG4-RD presents with heterogeneous clinical features, most commonly affecting the meninges, orbits, lacrimal and salivary glands, thyroid, lungs, pancreas, bile ducts, kidneys, retroperitoneum and aorta [9]. A latent class analysis of a large international patient cohort identified four subtypes: pancreato-hepato-biliary disease, retroperitoneal fibrosis and aortitis, Mikulicz’s syndrome with systemic involvement, and head-and-neck-limited disease [8]. These subtypes vary not only by the organs and regions involved but also by patient age, sex, ethnicity, and serum IgG4 levels [10].

The most frequent phenotype in our cohort was retroperitoneal fibrosis and/or aortitis (43%), while the least common phenotype was head and neck-limited disease (14%). Our findings contrast with those previously reported [8,10]. In the American cohort, Wallace et al. found pancreato-hepato-biliary disease to be the most frequent phenotype (31%), whereas the other three phenotypes (retroperitoneal fibrosis and/or aortitis, head and neck-limited disease and Mikulicz syndrome with systemic involvement) were almost equally prevalent (22% to 24% each) [8]. In a recent Chinese cohort, An et al. also reported pancreato-hepato-biliary disease as the most frequent phenotype (46.5%), while retroperitoneal fibrosis and/or aortitis was the least commonly observed (4.7%) [11]. These observed differences may reflect ethnic and geographic variations in the studied populations. As the number of patients in each phenotype group in our study was small, an analysis of potential variances between groups was not feasible. Regarding individual organ involvement, the three most frequently affected organs or body regions in our patients were the retroperitoneum (28%), pancreas (20%) and salivary glands (20%). Our results are consistent with an Italian study in which pancreas (41%), retroperitoneum (19%) and salivary glands (19%) represented the most frequently affected organs [12]. Slightly different findings were reported by Ebbo et al. in a multicentre French study, which identified lymph nodes (76%), pancreas (52%), salivary glands (44%) and kidneys (44%) as the most frequently involved organs [13].

Understanding pathogenetic mechanisms is fundamental for the development of effective treatment of IgG4-RD. At present, the genetic and environmental risk factors for IgG4-RD are still poorly understood, but both influence immune mechanisms that drive inflammation and fibrosis, leading to organ damage and dysfunction. B cells and T cells, in co-operation with fibroblasts and other inflammatory cells, play a key role in the disease process. It has been shown that the concentration of peripheral blood plasmablasts and memory B lymphocytes correlates with disease activity. B cells infiltrate tissues and secrete various inflammatory and profibrotic molecules. Patients with IgG4-RD typically have elevated serum IgG4 concentrations, which probably represent a compensatory response to chronic aberrant immune activation. In addition, B lymphocytes communicate closely with T lymphocytes. Several T cell subsets have been identified in IgG4-RD, for example, follicular helper T cells, and CD4+ and CD8+ cytotoxic T lymphocytes. While follicular helper T cells play a role in antibody switching (to IgG4 and IgE) and B cell activation, cytotoxic T lymphocytes contribute to tissue damage and fibrosis by secreting proapoptotic (e.g., perforin, granzyme and granulysin) and profibrotic molecules (such as transforming growth factor β, interleukin-1β and interferon γ). Immunohistochemical studies support the hypothesis of a spatiotemporal progression from the inflammatory to the fibrotic phase of the disease [1,14]. Alongside this, a classification into two disease phenotypes (predominately proliferative and predominantly fibrotic) has been recently proposed [2,3]. In our cohort, 63% of patients had the predominantly proliferative and 37% had the predominantly fibrotic phenotype.

Almost three-quarters of our patients fulfilled the 2019 ACR/EULAR classification criteria for IgG4-RD. These data are in line with the literature, despite the fact that not all our patients underwent an organ or tissue biopsy, and that assessing the performance of the classification criteria was not the main objective of this study [15,16,17].

Our research is not without limitations. Although the study covered a 13-year period, the number of IgG4-RD cases was low. The participation of both university centres in Slovenia would not only have increased the number of cases but would have enabled us a direct estimation of the incidence for the entire country. The retrospective design is commonly associated with incomplete and missing data, and this was also true for our study. In addition, data extraction focused on the presenting clinical features and initial management, and data on the long-term outcome are not presented. Furthermore, diagnostic and treatment approaches varied between departments, making the interpretation of the findings challenging. A standardised protocol would certainly improve the level of patient care.

We believe this study also has strengths. Multiple departments were involved, and valuable data were provided by the Institute of Pathology. Due to the high risk of miscoding, we did not use the International Statistical Classification of Diseases (ICD 10) coding system (which assigns D89 for IgG4-RD) to search electronic medical records for potential cases. Instead, we used the keyword “IgG4” to identify relevant cases. The number of total search results requiring evaluation was high, but we believe that this approach reliably detected patients who had not undergone histopathological diagnostics.

To conclude, this study investigated for the first time the incidence rate and clinical characteristics of IgG4-RD in Slovenia. It showed that, with an estimated incidence rate of 5 cases per million individuals, IgG4-RD is a relatively rare rheumatic disease, with the retroperitoneal fibrosis/aortitis phenotype predominating in our cohort.

## 5. Key Messages

- Epidemiological data on IgG4-RD are globally scarce.
- The incidence of IgG4-RD in Slovenia is 5 cases per million.
- Retroperitoneal fibrosis-aortitis phenotype predominated in our cohort.

## Figures and Tables

**Table 1:** Estimated Incidence Rate of IgG4-RD by Year.

Year	Adult Residents of Ljubljana Region	IgG4RD	Incidence Rate (95% CI) Per 10^6^
**2012**	528,561	1	1.89 (0.05; 10.54)
**2013**	531,233	1	1.88 (0.05; 10.49)
**2014**	533,761	2	3.75 (0.45; 13.54)
**2015**	535,101	5	9.34 (3.03; 21.81)
**2016**	535,946	2	3.73 (0.45; 13.48)
**2017**	537,297	2	3.72 (0.45; 13.45)
**2018**	538,116	2	3.72 (0.45; 13.43)
**2019**	543,932	3	5.52 (1.14; 16.12)
**2020**	549,121	2	3.64 (0.44; 13.16)
**2021**	550,287	5	9.09 (2.95; 21.20)
**2022**	548,170	4	7.30 (1.99; 18.68)
**2023**	552,946	5	9.04 (2.94; 21.10)
**2024**	556,912	1	1.80 (0.05; 10.01)
**2012–2024**		**35**	**4.96 (3.46; 6.91)**

**Table 2:** Characteristics of IgG4-RD Patients.

Organ/Region Involvement	Number and % of Patients (Out of 35)
Meninges	1 (2.9%)
Orbital structures	6 (17.1%)
Salivary glands	7 (20.0%)
Lungs	2 (5.7%)
Pleura	3 (8.6%)
Hepatobiliary tract	4 (11.4%)
Pancreas	7 (20.0%)
Kidneys	3 (8.6%)
Omentum	4 (11.4%)
Retroperitoneum	10 (28.6%)
Aorta	6 (17.1%)
Lymph nodes	5 (14.3%)
**Laboratory Tests**	
Elevated serum IgG4	23/28 (82.1%)
Hypergammaglobulinemia	8/21 (38.1%)
Hypocomplementemia	1/17 (5.9%)
Peripheral eosinophilia	2/23 (8.7%)
**Biopsy Findings**	31/35 (88.6%)
Consistent with IgG4-RD	23/31 (74.2%)
Possible IgG4-RD	5/31 (16.1%)
Negative/non-representative	3/31 (9.7%)
**Fulfilment of 2019 ACR/EULAR Classification Criteria**	26/35 (74.3%)

## Data Availability

The datasets analysed in the current study are available from the corresponding author upon reasonable request.
